# Biomimetic electrospun scaffolds for engineered heart tissue: from design parameters to drug testing platforms

**DOI:** 10.3389/fbioe.2026.1711698

**Published:** 2026-02-10

**Authors:** Raminta Vaiciuleviciute, Aidas Alaburda, Ilona Uzieliene, Kornél Kistamás, Máté Lengyel, András Dinnyés, Christian Bergaud, Eiva Bernotiene

**Affiliations:** 1 Department of Regenerative Medicine, Innovative Medicine Centre, Vilnius, Lithuania; 2 Institute of Biosciences, Life Sciences Center, Vilnius University, Vilnius, Lithuania; 3 BioTalentum Ltd., Gödöllő, Hungary; 4 Department of Physiology and Animal Health, Institute of Physiology and Animal Nutrition, Hungarian University of Agriculture and Life Sciences, Gödöllő, Hungary; 5 Laboratory for Analysis and Architecture of Systems - French National Centre for Scientific Research (LAAS-CNRS), University of Toulouse, Toulouse, France; 6 Faculty of Fundamental Sciences, Vilnius Gediminas Technical University, VilniusTech, Vilnius, Lithuania

**Keywords:** biomimicry, drug testing, engineered heart tissue, heart-on-a-chip, nanofibers

## Abstract

Electrospun nanofibers have emerged as a promising platform for cardiac tissue engineering, offering unique opportunities to recapitulate the native myocardial extracellular matrix (ECM) architecture. This comprehensive review examines the critical design parameters affecting cardiomyocyte function and maturation on electrospun scaffolds, including fiber diameter, material composition, alignment, and pore architecture. Recent advances in conductive polymers and hybrid material systems have shown particular promise for enhancing electrical coupling and functional maturation of stem cell-derived cardiomyocytes. However, significant challenges remain in achieving complete cardiomyocyte maturation, particularly regarding calcium handling properties and metabolic characteristics. This review synthesizes current knowledge on technical characteristics of biomimetic nanofibrous scaffolds, identifying future directions for translating these approaches toward realistic cardiac models and potential clinical cardiac regenerative applications.

## 1 Introduction

Cardiovascular diseases (CVDs) remain the foremost cause of mortality worldwide, responsible for an estimated 17.9 million deaths annually and accounting for approximately 32% of all global deaths (WHO, 2025). The prevalence of CVDs continues to rise, with projections indicating a further increase in both incidence and associated healthcare costs in the coming decades (Chong et al., 2024). Despite the availability of numerous pharmacological treatments, many drugs show limited efficacy, variable patient responses, or cardiotoxic side effects that are often not detected in preclinical testing. Current 2D culture systems and animal models fail to fully recapitulate the structural and functional complexity of human myocardium, limiting their predictive value for drug safety and efficacy. These limitations highlight the urgent need for advanced *in vitro* models—such as engineered heart tissues based on biomimetic scaffolds—that provide physiologically relevant platforms for drug testing and disease modeling (Singh et al., 2024).

In response, cardiac tissue engineering has emerged as a transformative field, aiming to restore, maintain, or improve heart function by combining cells, biomaterials, and bioactive molecules (Razavi et al., 2024). Central to this approach is the development of biomimetic scaffolds - engineered structures designed to replicate the complex architecture, mechanical properties, and biochemical cues of native heart tissue. These scaffolds provide a supportive microenvironment that promotes the alignment, maturation, and function of cardiomyocytes (CMs), facilitates vascularization, and represents biomimicry of the myocardium by engineered heart tissue (EHT) (Khan et al., 2023; Kim M. et al., 2025; Marino et al., 2025).

Among various biomaterial platforms, electrospun nanofibers have gained significant attention due to their ability to mimic the nanoscale architecture of native cardiac extracellular matrix (ECM) and provide topographical cues for cell alignment and function (Zhao et al., 2015; Broadwin et al., 2024). Recent advances in scaffold design, including the use of natural and synthetic polymers, three-dimensional (3D) printing, and bioactive modifications, have significantly enhanced the ability to mimic the ECM and dynamic environment of the heart, thereby improving the prospects for myocardial repair and regeneration (Khan et al., 2025; Khanna et al., 2021; Razavi et al., 2024).

Beyond their therapeutic potential, biomimetic cardiac scaffolds are increasingly recognized as powerful platforms for drug testing and disease modeling. Traditional drug testing methods, such as animal models and two-dimensional (2D) cell cultures, often fail to accurately predict human cardiac responses due to species differences and the lack of physiological complexity (Bédard et al., 2020). In contrast, engineered cardiac tissues and scaffolds can recapitulate the electrophysiological, mechanical, and structural properties of native myocardium, providing a more physiologically relevant environment for assessing drug efficacy and toxicity (Zhao et al., 2020). The integration of cardiac scaffolds into drug development pipelines also addresses ethical concerns by reducing reliance on animal models and offers the potential for personalized medicine approaches, where patient-specific cells can be used to model individual responses to therapies. Furthermore, emerging technologies such as precision medicine, artificial intelligence, and real-time biosensing are increasingly being combined with engineered cardiac tissues to create comprehensive systems that support both therapeutic and testing applications (Deir et al., 2024; Zhao et al., 2020; Van Den Berg et al., 2019).

## 2 Biomimicry of human myocardium

Biomimicry is the practice of emulating nature time-tested patterns, structures, and strategies to make natural systems successful and applying these insights to human-made designs (Ahamed et al., 2022). In tissue engineering, biomimicry refers to the design and fabrication of materials, scaffolds, and systems that replicate the structure, function, and microenvironment of native tissues, such as heart (Bolonduro et al., 2020). The goal is to create engineered tissues that closely resemble their natural counterparts in terms of architecture, mechanical properties, biochemical cues, and physiological function (Pomeroy et al., 2020). This includes mimicking the ECM, which provides structural and biochemical support to cardiac cells, as well as reproducing the unique mechanical and electrical properties of the heart (Hendrickson et al., 2021).

### 2.1 Structural organization of human myocardium

**FIGURE 1:**
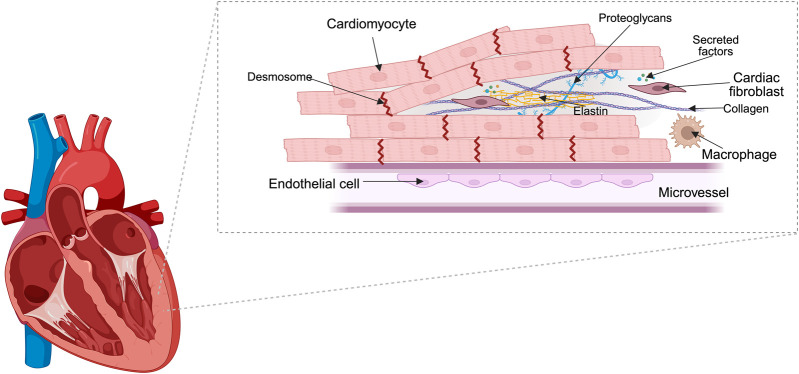
Structural organization of myocardium. Diagram showing the main cell types in cardiac muscle. Cardiomyocytes form interconnected contractile fibers, while fibroblasts occupy the interstitial space and maintain the ECM. Endothelial and smooth muscle cells are present in vascular structures, and resident immune cells are dispersed throughout the tissue.

Myocardium is composed of cardiac cells, including atrial or ventricular CMs, mural cells (smooth muscle cells and pericytes), CFs, endothelial cells (ECs) and immune (myeloid and lymphoid) cells and ECM (Litviňuková et al., 2020; Talman and Kivelä, 2018) (Figure 1). The cardiac ECM is a 3D network composed primarily of structural proteins such as glycoproteins, proteoglycans, and glycosaminoglycans. Fiber glycoproteins, such as collagens (mostly collagen I) and elastins, ensure the structure, while other nonstructural proteins, such as fibronectin, laminin, thrombospondin and others are important in ECM rearrangement, anchor growth factors [transforming growth factor-β (TGFβ), platelet-derived growth factor (PDGF) and fibroblast growth factor (FGF)], cytokines, chemokines, proteases (Rienks et al., 2014; Silva et al., 2021). The myocardium is highly anisotropic, meaning its fibers in ventricles are aligned in specific directions, while atrial fibers are multiple overlapping bundles running along different directions, which is essential for efficient contraction and force transmission (Piersanti et al., 2021; Hoermann et al., 2019). In native myocardium, cardiac muscle fibers are typically smaller in diameter than skeletal muscle fibers and are highly branched and interconnected (Shadrin et al., 2016). Cardiac myofibrils (the contractile elements within CMs) have diameters in the range of 1–2 μm, while ECM fibers in the heart, such as collagen, are typically in the 50–500 nm range, with some variability depending on the specific ECM protein and tissue region (Al-Khayat, 2013; Wittig and Szulcek, 2021).

CMs are interconnected cells which contract together synchronously. They are interconnected by intercalated discs, which include desmosomes, adherens junctions, and gap junctions, facilitating both mechanical and electrical coupling. Desmosomes and adherens junctions mechanically couple CMs together, gap junctions allow propagation of action potential from one cell to another (Noorman et al., 2009; Gutstein et al., 2003).

CFs produce, maintain and remodel ECM by producing collagens I and III and matrix metalloproteinases (MMP) and their inhibitors (tissue inhibitor of MMP; TIMP), also cardiac protective cytokines, such as IL-33, extracellular vesicles (EVs) in response to physiological stimuli (Nicin et al., 2022). During the injury or chronic disease CFs differentiate into myofibroblasts that secrete excessive amount of ECM proteins together with paracrine factors TGFβ, Angiotensin-II (Ang-II), and IL-6 leading to hypertrophy (Bowers et al., 2022; Krenning et al., 2010; Nicin et al., 2022). ECs regulate vascular tone, permeability in smooth muscle cells and contractile response of CMs through signaling molecules like nitric oxide (NO), endothelin-1 (ET-1) (Hsieh et al., 2006). Immune cells, such as macrophages contribute to homeostasis, repair, for instance, resident macrophages secrete anti-inflammatory cytokines, clear apoptotic cells, damaged mitochondria and even facilitate electrical conduction, while recruited macrophages during cardiac injury produce pro-inflammatory mediators and clear necrotic tissue (Chen et al., 2024; Hulsmans et al., 2017).

The heart is densely vascularized with an intricate capillary network to meet its high metabolic demands. Efficient oxygen delivery is critical for CM survival and function, as these cells rely heavily on aerobic metabolism. Replicating the vascular network is a major challenge in engineered tissues, as inadequate vascularization can lead to hypoxia and cell death (King et al., 2022; Liu Q. et al., 2025).

### 2.2 Electromechanical properties of human myocardium

The human heart operates under constant mechanical forces, which include both active and passive components. Active forces are generated by the contraction of CMs during each heartbeat, while passive forces arise from the elastic properties of the myocardial tissue and ECM (Knight et al., 2021). The ECM and the cellular architecture determine myocardial stiffness, elasticity, and the ability to withstand these forces, while mechanical cues from the ECM and hemodynamic loads regulate cardiac development, remodeling, and adaptation to stress. CM sarcomeres generate cyclic strain and stretch required to pump blood (Leite Coscarella and Kwon, 2025). One of the main parameters describing myocardial contraction is fractional shortening, which is about 10% in human myocardium and the average force is 51 ± 8 kN/m^2^ (Kvam et al., 1997; Van Der Velden et al., 1998).

Passive mechanical properties are largely defined by stiffness and elasticity. Stiffness describes the resistance to deformation under applied force, typically expressed in kilopascals (kPa). In healthy human myocardium, stiffness ranges from 1.7 kPa in diastole to 8.6 kPa in systole. Other studies report diastolic stiffness values of 8–15 kPa, which can increase significantly in pathological conditions: up to 25 kPa in hypertension and heart failure with preserved ejection fraction (HFpEF), and exceeding 55 kPa in myocardial scar tissue after infarction (Emig et al., 2021). These mechanical forces are not only essential for the immediate contractile function of the heart but also play a pivotal role in mechanotransduction—the process by which mechanical stimuli are converted into biochemical signals. Mechanoreceptors such as integrins and mechanosensitive ion channels detect alterations in the mechanical cues of the microenvironment and activate signaling cascades that regulate cell proliferation, differentiation, and ECM remodeling (Lindsey, 2014).

During heart development, initial contractility in the early cardiac tube and progressive ECM deposition lead to increased stiffness during maturation (Del Monte-Nieto et al., 2020). This increase in stiffness enhances mechanical strain, promoting greater structural organization and interconnection within heart tissue (Gaetani et al., 2020; Silva et al., 2021). These developmental processes suggest that exposing CMs to mechanical cues resembling physiological conditions may enhance *in vitro* cardiac differentiation and maturation.

In the myocardium, electrical conductivity is mediated by the ionic cytoplasm of CMs and low-resistance gap junction channels, primarily connexin-43 (Cx43), which enable rapid propagation of action potentials across the tissue, while collagen functions as an electrical insulator rather than a conductor, serving primarily a structural and mechanical role (Ke et al., 2025; Ul Haq et al., 2021). The bulk longitudinal conductivity in myocardium is about 0.35–0.38 S/m (Stinstra et al., 2005). Electroconductive scaffolds could support cell–cell communication, transfer of action potential and synchronous beating in engineered tissues (Ul Haq et al., 2023).

Together, these findings underscore that matching the electromechanical environment of engineered cardiac tissues to physiological conditions, including stiffness, elasticity, and conductivity, is critical for promoting functional maturation, structural organization, and long-term performance of engineered myocardium.

### 2.3 Advantages of three-dimensional architectures

**FIGURE 2:**
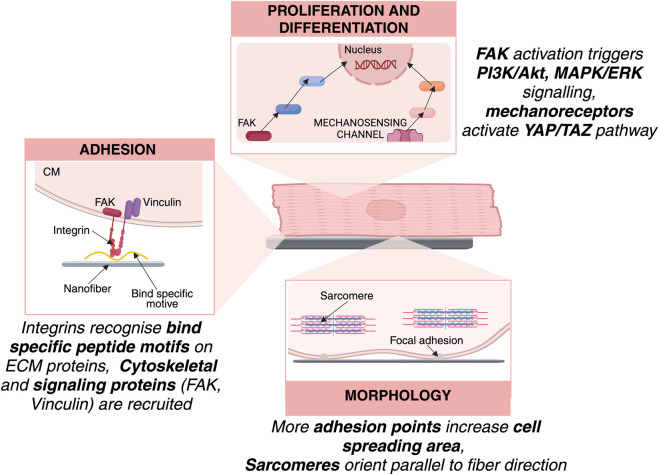
Cell–Scaffold interactions governing CM behavior. ECM proteins mediate integrin binding at the cell–scaffold interface, initiating focal adhesion assembly through focal adhesion kinase (FAK) and activation of signalling pathways. Substrate stiffness and topography regulate mechanotransduction pathways, influencing CM proliferation and morphogenesis.

3D scaffolds are designed to closely mimic the ECM of native cardiac tissue, providing a spatial arrangement that supports the natural organization and function of cardiac cells (Wang et al., 2021). In 2D cultures, cardiac cells typically exhibit a flattened morphology and form extensive focal adhesions with the substrate. While this setup is useful for basic studies, it does not accurately reflect the *in vivo* environment, where cells are surrounded by ECM and neighboring cells in three dimensions (Soares et al., 2012; Zuppinger, 2019). In contrast, the 3D environment promotes a more compact, physiologically relevant cell shape, increases cell-cell interactions, and facilitates the formation of multiple cellular junctions (Table 1; Zhang et al., 2022). This environment supports more natural cell adhesion and proliferation, leading to the formation of organized tissue structures that are essential for proper cardiac function (Figure 2) (Rogozinski et al., 2022).

**TABLE 1:** Summary of key practical and biological differences between 2D monolayer cultures and 3D cell-based models used in cardiac tissue engineering. The table highlights major aspects including cost, ease of handling, monitoring, reproducibility, screening potential, cell organization, cellular interactions, external stimulation, and physiological relevance.

Parameter		2D	3D	References
Complexity	Cost	Low	High	Gisone et al. (2022)
	Ease of use	Easy	More demanding handling	Gisone et al. (2022)
	Imaging	Easy	Limited	Gisone et al. (2022)
	Electrophysiology measurement	Simple to use patch clamp, sharp electrodes	Contractile force and kinetic measurements	Mohr et al. (2022)
	Reproducibility	High	Lower	Gisone et al. (2022)
	Screening	Rapid screening	Physiologically relevant screening	Krug et al. (2025)
	Relevance	Lower relevance	High	Krug et al. (2025)
Biological parameters	Cell alignment	Random cell alignment	Controlled/aligned architecture	Gisone et al. (2022), Mohr et al. (2022)
	Cell maturation	Immature phenotype	Enhanced maturation	Gonzalez et al. (2022)
	Cellular interactions	Limited interactions	Multicellular interactions	Gisone et al. (2022), Mohr et al. (2022)
	ECM deposition	Minimal ECM remodeling	Active ECM production	Mohr et al. (2022)
Functional parameters	External stimulation	More difficult to apply external mechanical or electrical stimulation	Enables physiological stimulation	Mohr et al. (2022)
	ECM deposition	Minimal ECM remodeling	Active ECM production	Duval et al. (2017)
	Oxygen/nutrient gradients	Uniform exposure	Limited diffusion	Gisone et al. (2022), Mohr et al. (2022)
	Mechanical properties	Non-physiological stiffness	Tissue-like stiffness	Sridharan et al. (2021)

3D nanofibrous heart-on-a-chip models can incorporate mechanical and electrical stimuli that are essential for the maturation and function of cardiac cells. These stimuli are difficult to replicate in 2D systems but are readily integrated into 3D architectures, leading to the development of tissues that contract and conduct electrical signals in a manner similar to native myocardium. This capability is vital for studying cardiac physiology, disease mechanisms, and drug responses in a clinically relevant context (Gu et al., 2024; Kieda et al., 2024).

Despite their advantages, 3D nanofibrous architectures also present certain challenges compared to 2D models. These include increased complexity in fabrication, higher costs, the need for specialized technical expertise, and difficulties in standardization and scalability (Srivastava et al., 2024). Visualization, imaging and assaying 3D tissues can also be more challenging due to issues with light and reagent penetration. Additionally, maintaining appropriate nutrient and oxygen gradients and permanent flow of medium imitating circulation within 3D constructs requires careful control of the microenvironment (Molina-Martínez and Liz-Marzán, 2022). Inadequate scaffold design with suboptimal parameters, can limit maturation, as demonstrated in polycaprolactone (PCL)–gelatin nanofibrous scaffolds where inadequate stiffness and reduced cell–cell interactions resulted in maturation levels comparable to 2D cultures (expression of NKX-2.5 and α-sarcomeric actinin) (Sridharan et al., 2021).

In summary, 3D nanofibrous heart-on-a-chip models offer substantial benefits over traditional 2D systems by providing a more physiologically relevant environment that supports enhanced cell behavior, tissue maturation, and functional integration. These advantages translate into more accurate models for cardiac research, drug testing, and disease modeling, paving the way for improved clinical outcomes and the development of personalized therapies. However, the adoption of 3D models must be balanced with considerations of complexity, cost, and technical challenges, which continue to be areas of active research and development.

## 3 Heart tissue engineering principles

### 3.1 Biocompatibility

In heart tissue engineering, biocompatibility is defined as the ability of a scaffold or construct to interact with the host environment without eliciting toxic or immunogenic responses, while supporting essential cellular processes such as adhesion, proliferation, differentiation, and functional maturation.

Mechanistically, scaffold surface chemistry and nano/microarchitecture play pivotal roles in both adhesion and proliferation. Plasma treatment introduces hydroxyl (–OH), ether (C–O), and ester (O=C–O) groups to polymeric scaffolds, increasing surface hydrophilicity and providing anchoring points for ECM proteins such as fibronectin and laminin, which mediate integrin binding and promote adhesion, spreading, and proliferation (Léger et al., 2024). CMs express integrins that bind specific peptide motifs (e.g., RGD) on ECM proteins, such as fibronectin, laminin, forming focal adhesion. Focal adhesion kinase is activated and triggers signalling pathways, regulating cell proliferation and differentiation (Israeli-Rosenberg et al., 2014). For example, polar/hydrophobic/ionic polyurethane (D-PHI) scaffolds have been shown to enhance human induced pluripotent cell-derived cardiomyocyte (hiPSC-CM) alignment and proliferation, creating anisotropic cellular organization that is critical for functional impulse propagation and contractility (Ding et al., 2020; Chen et al., 2022).

Biocompatible scaffolds should support physiological CM morphology, characterized by elongation, anisotropic alignment, and organized sarcomere structure. For instance, hiPSC-CMs cultured on aligned poly (lactic-co-glycolic acid) (PLGA) nanofibers became elongated and parallelly oriented within 7 days, closely resembling native ventricular myocytes (Khan et al., 2015). Such structural alignment enhances gap junction formation (e.g., Cx43) and supports uniform electrical conduction.

Scaffold architecture can also direct cardiogenic iPSC differentiation through mechanotransduction pathways. Nanoscale fibers mimic the stiffness and geometry of the native ECM, activating integrin-mediated focal adhesion complexes and YAP/TAZ signaling, which regulate lineage-specific gene expression. Electrospun coaxial PCL-gelatin nanofibrous scaffolds increased expression of SIRPA and ISL-1, which means it promotes differentiation of hiPSCs to cardiac progenitors (Sridharan et al., 2021). Reduced graphene oxide (rGO)/poly (lactide-co-ε-caprolactone (PLCL) nanofibers also support hiPSC cardiac differentiation reaching more than 80% of troponin T (TnT) positive cells (Tan et al., 2023). Electrospun 3D nanofibrous PCL scaffolds have been shown to promote iPSC differentiation toward cardiac phenotypes, with increased expression of atrial natriuretic factor (ANF) and myosin light chain (MLC) isoforms MLC2a (atrial) and MLC2v (ventricular) (Zhang et al., 2022).

*In vitro* studies increasingly demonstrate that biomaterial–immune interactions affect the response of engineered cardiac tissues. When nanofiber scaffolds are seeded with macrophages or exposed to immune-conditioned media, scaffold properties—including surface chemistry, topography, fiber diameter, stiffness, orientation, and protein adsorption—can direct macrophage polarization toward either pro-inflammatory (M1) or pro-regenerative (M2) phenotypes (Thilakan et al., 2025; Salthouse et al., 2023). Surface chemistry plays an important role: neutral and positively charged groups (–NH_3_^+^, –OH) have been shown to reduce immune activation by limiting immune cell adhesion, while negatively charged groups (–COOH) can suppress immune cell infiltration and inflammatory responses, particularly when combined with appropriate topographical cues (Amani et al., 2024). Nanofiber architecture further modulates immune behavior; for example, highly aligned PLLA nanofibers inhibited LPS-induced macrophage M1 polarization and promoted an M2 phenotype *in vitro* (Xie et al., 2022). Increased surface roughness of PCL nanofibers enhanced protein and damage-associated molecular pattern adsorption, altering macrophage cytokine release profiles *in vitro* (Alemifar et al., 2024).

Emerging AI-based models further support rational scaffold design by predicting inflammatory responses, such as macrophage TNF-α production, based on nanofiber parameters including fiber diameter, pore size, and surface ruffling index (Sujeeun et al., 2025). Beyond passive material tuning, bioactive immune-modulating strategies are feasible: M2 macrophage membrane–coated PCL nanofibers suppressed TNF-α and IL-1β activity *in vitro*, demonstrating anti-inflammatory effects while supporting tissue remodeling (Nakkala et al., 2022).

Collectively, these studies demonstrate that nanofiber scaffolds can function not only as structural supports for CMs, but also as immune-instructive platforms that minimize inflammatory signaling and improve the reproducibility and reliability of engineered heart tissues for drug safety and efficacy testing.

In summary, scaffold biocompatibility extends beyond non-toxicity to actively supporting adhesion, proliferation, differentiation, and maturation of CMs. These responses are essential for creating EHT that faithfully replicate native myocardial structure and function.

### 3.2 Structural mimicry

Biomimetic scaffolds are designed to imitate the fibrous, anisotropic (directionally dependent) structure of the heart ECM. Techniques such as electrospinning and 3D bioprinting are used to fabricate scaffolds with aligned fibers and microchannels that guide cell organization and tissue formation.

#### 3.2.1 Fiber diameter

The diameter of electrospun nanofibers significantly influences CM behavior and function. Research has consistently demonstrated that fiber diameter affects cell attachment, proliferation, and differentiation outcomes. Electrospun scaffolds that aim to mimic the ECM of the myocardium often use fiber diameters in the 100–500 nm range to replicate the nanoscale architecture of native collagen fibers, while for mimicking the alignment and organization of myofibrils, fiber diameters of 1–2 μm can be used to guide CM orientation and function (Wittig and Szulcek, 2021). Poly (lactide-co-glycolide) (PG) nanofibrous scaffolds with smaller fiber diameters (239 ± 37 nm for random fibers and 269 ± 33 nm for aligned fibers) have been compared to PCL scaffolds with larger diameters ranging from 450 to 800 nm (average 565 ± 30 nm). The evidence indicated that smaller diameter fibers (200–300 nm) generally promote cell attachment and proliferation compared to larger diameter fibers (500–800 nm) (Kai et al., 2011). This observation aligns with the biomimetic principle that CMs preferentially interact with substrates that recapitulate the nanoscale architecture of native ECM, thereby maintaining cytoskeletal dynamics characteristic of early-stage cardiac cells.

#### 3.2.2 Fiber alignment

Fiber alignment is a critical scaffold design parameter that profoundly influences CM organization, morphology, and functional performance (Kristen et al., 2020). In the native myocardium, CMs are embedded in an anisotropic ECM, where collagen and elastin fibers are directionally aligned to optimize force transmission and electrical conduction (Cyr et al., 2023). Mimicking this structural anisotropy in engineered scaffolds helps reproduce native tissue architecture and function.

HiPSC-CMs grown on anisotropic plasma-treated PCL/chitosan (CS) nanofibers exhibit elongated, rod-like morphology and parallel alignment, closely mimicking native ventricular myocytes. In contrast, cells on random fibers often display a star-like morphology with radial spreading. HiPSC-CMs cultured on aligned fibers had greater sarcomere spacing and reduced nucleus circularity compared to those on random fibers (Léger et al., 2024). Structural alignment translates into measurable functional advantages. HiPSC-CMs cultured on aligned PCL scaffolds showed higher and more consistent frequencies of synchronous spontaneous contraction, along with increased MYH7 expression and a higher β-MyHC/α-MyHC ratio (Liu et al., 2023). Similarly, aligned carbon nanofiber (CNF)/PLGA scaffolds supported greater cell density and elevated expression of cardiac markers including cardiac TnT, Cx43, and α-sarcomeric actin compared to random fibers (Webster et al., 2014). These effects are mediated by alignment-induced changes in integrin binding and cytoskeletal organization, which enhance gap junction formation and anisotropic conduction pathways. While anisotropic scaffolds effectively induce cell alignment, improve sarcomere organization, and increase cardiac gene expression, alignment alone does not ensure full CM maturation. Aligned electrospun fibers had limited effects on calcium handling even if mRNA expression of key functional maturation markers (TNNT2, MYL7, MYH6, TTN) and ion channels (ATP2A2, CASQ2, HCN1, KCNJ2 and KCNA4) was increased, suggesting that alignment must be combined with electrical/mechanical stimulation, biochemical cues, or metabolic conditioning to achieve adult-like CM function (Han et al., 2016).

Aligned fibers also modify surface physicochemistry and mechanical anisotropy. For example, in CNF/PLGA composites, fiber alignment increased hydrophobicity (measured by contact angle) and improved ECM protein adsorption compared to randomly oriented fibers. Alignment also altered fiber diameter distribution—aligned PCL/CS fibers were thinner (most in the 250–350 nm range) than random fibers (450–550 nm range). Furthermore, mechanical testing revealed that vertical Young’s modulus was higher in aligned CNF/PLGA fibers (7.90% ± 1.8%) compared to random (5.05% ± 1.1%), while the horizontal modulus was lower in aligned fibers (2.14% ± 0.3%) *versus* random (4.96% ± 1.3%), reflecting anisotropic stiffness (Webster et al., 2014). Notably, aligned Polyurethane (PU)/CS/Carbon nanotubes (CNT) scaffolds had lower resistance (higher conductivity) than their random-fiber counterparts, likely due to more continuous conductive pathways along the fiber axis (Ahmadi et al., 2021). The architecture of nanofiber scaffolds can directly influence vascular ingrowth. For example, hybrid fibrinogen–polylactic acid (FBG–PLA) nanofibers promoted oriented migration of endothelial cells and guided vessel-like structure formation (Gugutkov et al., 2017).

Fiber alignment regulates CM behavior through contact guidance–mediated mechanotransduction, constraining cell spreading and promoting cytoskeletal organization along a single axis. In conductive scaffolds, aligned fibers also facilitate continuous charge along the fiber axis, reducing electrical resistance. However, because alignment primarily affects structural and junctional organization, it must be combined with electrical, mechanical, or metabolic cues to achieve fully mature calcium handling and adult-like CM function.

#### 3.2.3 Pore size and scaffold architecture

In cardiac tissue engineering, pore size and porosity are key architectural parameters that control scaffold performance. They determine the efficiency of nutrient and oxygen diffusion, waste removal, and the ability of cells to migrate and infiltrate deep within the structure. These processes are particularly important for CMs, which have high metabolic demands and limited tolerance to hypoxia. At the same time, pore design must preserve mechanical integrity so the scaffold can withstand repetitive cardiac loading without collapsing (Mukasheva et al., 2024). While aligned electrospun nanofibers effectively recapitulate myocardial anisotropy and improve CM alignment and coupling, dense nanofibers alone often limit cell infiltration and do not guarantee perfusion (Zhuang et al., 2025).

In native human myocardium, ECM pores range from 10 to 50 μm, a size sufficient to accommodate CMs and small coronary vessels. CMs make up ∼70% of myocardial volume, and the ECM provides the structural framework for both mechanical force transmission and electrical signal propagation (Mukasheva et al., 2024). This natural architecture serves as a functional benchmark for engineered scaffolds. While aligned electrospun fibers are beneficial for directing CM anisotropy and improving electrical conduction, they often form compact structures that reduce pore size and hinder deep cell infiltration (Flaig et al., 2024), but in PCL scaffold aligned fibers had significantly higher overall porosity (Safaeijavan et al., 2014). Silk fibroin/carbon nanofiber scaffolds with 69%–71% porosity (pore diameter ∼78 μm) degraded faster enzymatically and promoted greater murine iPSC proliferation compared to 79% porosity scaffolds with larger pores (37–392 μm). However, after cardiac differentiation, higher-porosity, larger-pore scaffolds yielded higher TNNT2 and Nkx2.5 expression, indicating improved late-stage cardiac phenotype acquisition. However, the results with human cells are still lacking (Tufan et al., 2023).

An increasing number of studies employ hierarchical scaffold architectures that integrate nanofibers and microfibers to simultaneously address anisotropy, mechanical support, and mass transport limitations. At the nanoscale, aligned or patterned nanofibers provide contact guidance and anisotropic mechanical cues that regulate cell orientation, cytoskeletal organization, and electrical coupling. At the microscale, microfibers and strut-like features contribute structural integrity while generating interconnected pores and perfusable channels, facilitating oxygen and nutrient transport and supporting vascular network formation (Yeo and Kim, 2020). Representative examples highlight the versatility of this approach. Uniaxially micropatterned PCL/collagen struts combined with highly aligned electrospun alginate nanofibers were used to create hierarchical scaffolds for engineered skeletal muscle, demonstrating how microscale architecture can be combined with nanoscale alignment to direct tissue organization (Yeo and Kim, 2020). In a cardiac context, stacked PCL nanofiber mat layers were used to orient HL-1 CMs in a manner that mimics the transmural fiber rotation from endocardium to epicardium, illustrating how layered nanofiber architectures can reproduce native myocardial organization (Eom et al., 2021). Beyond electrospinning alone, advanced bioprinting techniques are increasingly integrated with nanofiber scaffolds to generate hierarchical vascular networks, offering a potential solution to the long-standing challenge of achieving adequate perfusion in thick cardiac constructs (Xing et al., 2023; Baghersad et al., 2023).

To overcome limitations of sufficient porosity and mechanical integrity, several fabrication strategies have been developed (Table 2).

**TABLE 2:** Representative fabrication strategies used to optimize scaffold porosity while preserving mechanical stability in electrospun and hybrid cardiac tissue engineering platforms. The table summarizes different architectural approaches, associated scaffold materials, and resulting pore characteristics, together with reported biocompatibility outcomes.

Fabrication strategy	Scaffold materials	Result	Biocompatibility	References
Combination electrospinning/electrospraying	PLLA with poly (glycerol sebacate)/cyclodextrin	Produced scaffolds with interconnected pores from tens to several hundred microns	Support adhesion and proliferation of human ventricular CFs	Flaig et al. (2024)
Material blending and structural tuning	Collagen/HA/polyaniline (PANi)	Porosities of 60% (aligned) and 64% (random), but swelling during culture reduced porosity over time	-	Roshanbinfar et al. (2019)
Coaxial fiber designs	PCL–gelatin	Scaffolds with 82.5% ± 6.2% porosity and interfiber spacing of 21.6 ± 6.74 μm	Supported hiPSC differentiation into cardiac progenitors, cell morphology similar to 2D cultures but enhanced cell–cell contact and paracrine signaling	Sridharan et al. (2021)
The Oriented Thermally Induced Phase Separation (OTIPS)	Carbohydrate template within a CS/collagen scaffold	Generated a microporous network within a scaffold	Supported synchronized beating under electrical stimulation and calcium transients in neonatal rat CMs	Fang et al. (2019)

Overall, optimal fiber diameter, alignment and pore size are critical for cell attachment, alignment and balancing nutrient transport, together with mechanical stability in cardiac scaffolds.

### 3.3 Scaffold materials and functional mimicry

Selecting scaffold materials for electrospun platforms is not merely a matter of biocompatibility and degradability. In cardiac tissue engineering, the material system must recreate the structural anisotropy, viscoelastic compliance, electrical percolation, and biochemical signaling of the native myocardial ECM (Sharma et al., 2019). The current consensus is shifting from single-component polymers to multifunctional hybrids that integrate synthetic backbones, natural bioactive phases, and conductive elements to enable functional mimicry of the beating myocardium.

#### 3.3.1 Synthetic polymer backbones: Structure, durability, and processing latitude

PCL and PLGA remain the workhorses for electrospinning because they combine robust processability with tunable mechanics and degradation. PCL maintains mechanical integrity under cyclic loading but is hydrophobic and degrades slowly, which can limit early cell adhesion and timely tissue remodeling; PLGA is comparatively more hydrophilic with faster hydrolysis into lactic/glycolic acids, which can favor initial cell attachment and spreading but reduce long-term structural persistence (necessitating blending or architectural reinforcement). These trade-offs are highlighted in comparative electrospinning studies and reviews focused on cardiovascular applications, which consistently recommend blending PCL/PLGA with bioactive or conductive phases to overcome single-polymer limitations and reduce the use of toxic solvents (Vogt et al., 2019).

#### 3.3.2 Natural polymers: Built-in bioactivity for integrin engagement

Natural phases—gelatin, collagen, and fibrin—introduce integrin-binding motifs (e.g., RGD - Arg–Gly–Asp) that catalyze focal adhesion signaling, cytoskeletal alignment, and sarcomere formation. However, their rapid enzymatic degradation and lower tensile strength typically require reinforcement by synthetic backbones (e.g., PCL- or PLGA-containing blends) to withstand repeated contractile cycles (Ahamed et al., 2022; Zhao et al., 2015). Recent coaxial electrospinning strategies place natural polymers in the shell or core to decouple mechanical support from bioactivity and to host releasable cargo. For example, coaxial PCL/gelatin nanofiber membranes have been fabricated to co-deliver small molecules from the shell while maintaining a mechanically competent substructure—an approach that readily translates to cardiac constructs where simultaneous structural guidance and biochemical priming are desirable. CS provides antimicrobial properties and can be modified to enhance conductivity when combined with conductive materials (Tamo et al., 2025). Silk fibroin has also emerged as a particularly promising natural polymer due to its exceptional mechanical strength and tunable degradation properties (Tufan et al., 2023).

##### 3.3.2.1 Growth factor incorporation

To effectively recreate the native myocardial microenvironment, biomimetic cardiac scaffolds should incorporate bioactive molecules such as growth factors - Vascular endothelial growth factor (VEGF), Epidermal growth factor (EGF), FGF, insulin-like growth factor (IGF), PDGF, and ECM-derived peptides. These molecules orchestrate critical signaling pathways that regulate cell survival, adhesion, proliferation, differentiation, and functional integration (Brown G. S. et al., 2023; White and Chong, 2020). Growth factor-binding polymers, such as heparin mimetic peptides, can efficiently bind and release growth factors (Mammadov et al., 2012). VEGF is one of the most extensively studied angiogenic signals in scaffolds. For instance, VEGF immobilized on electrospun poly-L-lactic acid (PLLA) nanofibers promoted endothelial cell infiltration and proliferation, upregulated endothelial marker CD31, while sustained release of heparin from the scaffold reduced proliferation of human coronary artery smooth muscle cells and platelet activation, thereby supporting vascular specificity and biocompatibility (Guex et al., 2014; Kang et al., 2022). Similarly, a nanofibrous PLCL/poly (2-ethyl 2-oxazoline) (PEOz) matrix loaded with VEGF and FGF induced endothelial cell morphological changes such as lamellipodia and filopodia formation, and increased expression of angiogenic receptor genes (VEGFR, FGFR, PDGFR) in HUVECs (Lakshmanan et al., 2016). These effects highlight how nanofiber scaffolds can be used to spatially present growth factors, activating downstream MAPK/ERK and PI3K/Akt signaling to promote angiogenesis and tissue integration.

##### 3.3.2.2 ECM proteins and peptides

In addition to soluble growth factors, the ECM provides essential biochemical cues through integrin-mediated adhesion. Incorporation of collagen, laminin, fibronectin, nephronectin, or ECM-derived peptide motifs such as RGD into nanofiber scaffolds enhances cell attachment and spreading by directly engaging integrin receptors and activating focal adhesion kinase (FAK) signaling. For example, RGD-modified scaffolds—including Ac-GRGD peptides or recombinant spider silk proteins (eADF4(C16))-RGD—improved CM adhesion, elongation, and alignment (Tallawi et al., 2015; Kumar et al., 2023). To date, many of these ECM-functionalized nanofiber scaffolds [e.g., GO/PLGA, poly (ester-urethane) urea] have been primarily investigated for vascular tissue engineering and could enhance vascularization of tissue and holds strong potential of translation into cardiac application (Shin et al., 2017; Zhu et al., 2017).

##### 3.3.2.3 Controlled release strategies

Another approach to biochemical mimicry is embedding controlled-release systems within nanofiber scaffolds. Nanocarriers such as PLGA nanoparticles, hyaluronic acid (HA) microspheres, lipid nanoparticles, inorganic nanoparticles, or EVs can provide localized, sustained delivery of growth factors and cytokines. This controlled release mimics the spatiotemporal signaling patterns of the native myocardium, extending the bioactivity of otherwise short-lived proteins and minimizing systemic side effects (Gil-Cabrerizo et al., 2023; Karnwal et al., 2024).

While these strategies have been widely studied in other regenerative contexts, their application in cardiac tissue engineering remains underdeveloped, representing a promising future research direction.

#### 3.3.3 Electroconductive materials for enhanced electrical coupling

Because excitation–contraction coupling relies on rapid impulse propagation and low-resistance cell–cell coupling, adding electronic/ionic conductivity to scaffolds can accelerate synchronization and maturation of hiPSC-CMs (Burnstine-Townley et al., 2020; Elkhoury et al., 2024).

Conductive polymers represent a significant advancement in cardiac tissue engineering, addressing the critical requirement for electrical coupling between CMs. The benefits of conductive scaffolds include enhanced electrical signal propagation, improved synchronous beating and better cell-cell communication leading to increased functional maturation (Zhang et al., 2023). Research has shown that increasing conductivity in fibers induces cell elongation and ECM synthesis (Liu et al., 2016). This demonstrates the importance of precise conductivity tuning for achieving optimal CM function.

Electrospun or hydrogel-based systems containing polyaniline (PANi), polypyrrole (PPy), or poly (3,4-ethylenedioxythiophene) (PEDOT): polystyrene sulfonate (PSS) provide percolative pathways that (i) reduce activation latency, (ii) support higher conduction velocities under pacing, and (iii) improve Ca^2+^-handling phenotypes compared with non-conductive controls (Parchehbaf-Kashani et al., 2020). In hiPSC-CM models, conductive blends created by electrospinning have been shown to improve structural organization and electrical coupling relative to inert counterparts, underscoring that conductivity is an *active* regulator of phenotype rather than a passive property (Gonzalez et al., 2023).

Importantly, progress has moved beyond *in vitro* readouts to disease-relevant function. A collagen–PEDOT:PSS hydrogel recently demonstrated antiarrhythmic efficacy *in vivo* and supported hiPSC-CM function, providing strong translational evidence that soft, conductive biomaterials can stabilize post-infarct conduction while remaining cell-compatible—a design principle equally applicable to electrospun constructs that incorporate PEDOT:PSS domains or coatings (Roshanbinfar et al., 2024). A number of recent reviews now codify design rules for conductive scaffolds—electrospun fibers included—covering dopant chemistry, percolation thresholds, hydration-dependent transport, and process–structure–property relationships necessary to achieve stable, physiological conductivity without sacrificing degradability or cytocompatibility. These analyses converge on hybrid strategies (e.g., PEDOT:PSS-gelatin, PPy- or PANi-functionalized protein matrices) that synergize ionic and electronic conduction within hydrated cardiac microenvironments (Furlani et al., 2023).

However, scaffold does not need to fully resemble electrical conductivity of native myocardium because CM electrical coupling *in vivo* is mediated by ionic conduction through cytoplasm and low-resistance pathways provided by connexin-based gap junctions. Researchers have developed scaffolds containing PEDOT:PSS/Polyvinyl alcohol (PVA) with specific crosslinking protocols to optimize conductivity and stability (Gonzalez et al., 2023).

CNTs and graphene derivatives (rGO, GO) are frequently embedded within polymer fibers to establish long, tortuous conduction pathways at low loading, while simultaneously contributing nanoscale roughness that promotes sarcomeric alignment (Roshanbinfar et al., 2019). A representative study produced aligned rGO/PLCL electrospun membranes that guided hiPSC-CM orientation and improved synchronization of calcium oscillations under electrical stimulation—evidence that combining anisotropy with conductivity can deliver “conduction-consistent” cardiac patches suitable for drug screening and disease modeling (Tan et al., 2023). Hybrid electroconductive designs have also progressed in the hydrogel domain [e.g., Gelatin Methacrylate (GelMA)–CNT], where aligned CNT architectures increase anisotropic conduction and foster synchronous beating (Ahadian et al., 2016).

However, higher conductivity improves electrical coupling and it can be enhanced by incorporating conductive fillers (Table 3).

**TABLE 3:** Overview of conductive fillers incorporated into electrospun and hybrid scaffolds to enhance electrical coupling in engineered heart tissue platforms. The table summarizes the type and concentration of conductive materials, corresponding scaffold matrices, resulting changes in electrical or physical properties, and reported biocompatibility outcomes.

Material	Percentage	Scaffold	Physical effect	Biocompatibility	References
Carbon nanotubes (CNT)	-	PU/CS	Significantly reduced electrical resistance	-	Ahmadi et al. (2021)
	1%	CS-PVA	-	Increased expression of cardiac markers in mesenchymal stem cells Nkx2.5, Troponin I, and β–MHC after 10 and 20 days of differentiation	Mombini et al. (2019)
Carbon quantum dots (CQD)	0.5% and 1%	Poly glycerol sebacate/PCL	Decreased fibers and increased wettability compared to PCL	Reduced mouse myoblast viability	Rastegar et al. (2021)
Reduced graphene oxide (rGO)	Up to 4%	PLCL	Decreased resistance	Improved the synchronization of calcium oscillations under electrical stimulation in hiPSC-CMs	Tan et al. (2023)
Polianiline (PANi)	-	CS	Smaller fibers. Aligned fibers had 91% higher electrical conductivity compared to random	-	Gu et al. (2018)
Polypyrrole (PPy)	10%–15%	CS and collagen	Highest conductivity with 15% PPy	10% PPy scaffolds had better biocompatibility tested with human skin fibroblasts	Zarei et al. (2021)

In recent years, electrical stimulation during *in vitro* culture, implemented with electrically conductive scaffolds, has emerged as a powerful tool in cardiac tissue engineering and is bringing the field closer to optimal artificial cardiac constructs (Scott et al., 2022).

## 4 Cardiomyocyte maturation strategies

### 4.1 Maturation challenges in hiPSC-CMs

HiPSC-CMs have become versatile tools for cardiovascular research, serving roles in disease modelling, cardiotoxicity testing, and heart-on-a-chip (HoC) platforms, largely due to their scalability and patient-specific origin (Lu et al., 2025). Despite these advantages, hiPSC-CMs generated using standard differentiation protocols exhibit a fetal-like phenotype that limits their translational relevance. Structurally, these immature cells display circular morphology, short and disorganized sarcomeres, low myofibril density, absence of T-tubules, and the predominance of fetal sarcomeric isoforms such as TNNI1, whereas adult CMs are rod-shaped, hypertrophic, polyploid, with aligned sarcomeres and express mature markers including MYH7, TNNI3, connexin-43, and TNNT2B (Kistamás et al., 2024).

This structural immaturity is compounded by metabolic and electrophysiological limitations. HiPSC-CMs primarily rely on glycolysis for energy, in contrast to adult CMs, which utilize oxidative phosphorylation and fatty acid β-oxidation to meet the high ATP demands required for efficient contraction (Cha et al., 2025; Barreto-Gamarra and Domenech, 2025). Electrophysiologically immature hiPSC-CMs exhibit spontaneous depolarizations, unstable resting potentials, and irregular contraction patterns, whereas mature cells maintain stable resting potentials around −80 to −90 mV and propagate synchronized action potentials (Hoekstra et al., 2012; Song et al., 2022; Lu et al., 2025). Nevertheless, immature hiPSC-CMs retain utility for studying neonatal physiology, early-onset cardiac disorders, and developmental toxicity, even if their incomplete maturation limits predictive power for adult disease modelling and pharmacological testing (Lamberto et al., 2023).

To address these challenges, a variety of strategies have been pursued, encompassing biochemical cues such as growth factors and fatty acids, biophysical stimuli including topographical, mechanical, and electrical signals, metabolic interventions, co-culture with supportive cell types like CFs, and targeted genetic modulation (Lu et al., 2025; Jang et al., 2025). Among these, scaffold-based approaches—particularly aligned nanofiber architectures—have emerged as a promising solution, providing structural alignment, mechanical support, and electrical guidance that collectively enhance maturation, improve predictive capacity for drug testing, and facilitate the development of functional tissue-engineered cardiac constructs (Zhang et al., 2025; Taylor et al., 2024; Wilson et al., 2025).

For broader overviews of biochemical, metabolic, and biophysical maturation strategies and realistic heart models in cardiotoxicity and disease modelling, see Table 4 and our recent reviews, (Kistamás et al., 2023; Kistamás et al., 2024).

**TABLE 4:** Strategies to overcome maturation challenges in hiPSC-CMs. Non-myocyte partners—including endothelial cells, fibroblasts, adipose-derived stromal cells, sympathetic neurons, and integrated microfluidic systems—provide complementary biochemical and biomechanical cues that collectively drive structural, electrophysiological, and metabolic maturation of CMs.

Maturation strategy	Immature hiPSC-CM features	Adult CM benchmark	Maturation strategy	Key outcomes	References
Structural	Circular shape, short/disorganized sarcomeres, fetal isoforms (TNNI1)	Rod-shaped, aligned sarcomeres, MYH7, TNNI3, Cx43, TNNT2B	Aligned nanofiber scaffolds, micropatterning	Elongation, sarcomere alignment, adult phenotype	Wu et al. (2025), Al Sayed et al. (2024)
Metabolic	Predominantly glycolytic ATP production, low mitochondrial content	Oxidative phosphorylation, FAO, high mitochondrial density	Fatty acid supplementation, ECM cues (collagen I), genetic/pharmacological modulation	Metabolic shift toward FAO, increased ATP production	Cha et al. (2025), Barreto-Gamarra and Domenech (2025)
Electrophysiological	Spontaneous depolarizations, unstable resting potentials, irregular contractions	Stable resting potential (−80 to −90 mV), synchronised APs	Conductive nanofibers (PEDOT:PSS, rGO, gold nanorods, MXene), mechanical/electrical training	Improved conduction, synchronised contractions, enhanced ion channel expression	(Jang et al., 2025) Hoekstra et al. (2012)
Biophysical cues	Poor alignment, weak contractility, poor force generation	Synchronous contraction, aligned sarcomeres, strong force generation	Substrate stiffness, cyclic stretch, strain-responsive substrates	Enhanced sarcomere alignment, calcium handling, conduction velocity	Shen et al. (2017), Pohjolainen et al. (2022)
Biochemical cues	Limited growth factors, hormones, and ECM composition, leading to poor adhesion, incomplete isoform switching, and glycolytic preference	Hormones, fatty acids, and ECM proteins drive adhesion, contractile protein maturation, oxidative metabolism, and coordinated excitation–contraction coupling	Growth factors, fatty acids, ECM proteins, peptide tethering	Upregulation of adult markers (MYH7, TNNI3, Cx43), improved Ca^2+^ handling and contractile force	Barreto-Gamarra and Domenech (2025), Lamberto et al. (2023)
Genetic/Pharmacological	Persistence of fetal isoforms (e.g., TNNI1), fetal-like and heterogeneous gene expression patterns with partial adult marker induction	Adult isoform expression (MYH7, TNNI3, connexin-43), stable and reproducible adult-like transcriptional profile	Overexpression of maturation drivers (CCND2, Cfp1), small molecules (3i-1262)	Enhanced sarcomere development, transcriptional remodeling, functional readouts, gene expression changes	Li et al. (2024), Pohjolainen et al. (2024)
Co-culture	Monocultures lack supporting cells, educed paracrine signaling, limited ECM remodeling, poor vascularization, absence of electromechanical coupling cues	Native myocardium contains multiple non-myocyte populations providing structural support, paracrine and juxtacrine signals, ECM turnover, vascularization, and autonomic regulation	Co-culture with endothelial cells, fibroblasts, stromal cells, neurons	Improved sarcomere organization, contractility, conduction, adrenergic response	Helle et al. (2021), Brown et al. (2024), Liu et al. (2024), Mohammadi et al. (2025)

### 4.2 Scaffold-based maturation enhancement

A major challenge in cardiac tissue engineering is achieving electrical, mechanical, and metabolic maturation of hiPSC-CMs. Biocompatible scaffolds can provide structural and biochemical cues that promote sarcomeric organization, metabolic shifts toward oxidative phosphorylation, and enhanced excitation–contraction coupling. Functional maturation metrics include increased cell area, sarcomere length, Cx43 expression, β-myosin heavy chain (MHC)/α-MHC ratio, MLC2v/MLC2a ratio, higher Ca^2+^ transient amplitude, and maximal upstroke velocity. Electrospun 3D nanoporous PCL scaffolds significantly enhanced these parameters compared to 2D cultures (Zhang et al., 2022).

Aligned nanofiber scaffolds fabricated from PCL or PU with tuned elasticity have been shown to promote CM elongation, enhanced sarcomere organization, and upregulation of key cardiac markers within just 10 days of culture (Wu et al., 2025; Maaref et al., 2024; Al Sayed et al., 2024). By creating suspended and oriented architectures that mimic the ECM, these scaffolds improve contraction synchrony, tissue alignment, and overall functional maturation compared with conventional 2D culture systems (Zhang et al., 2025; Taylor et al., 2024). Micropatterned substrates further refine maturation at the molecular level, driving adult ventricular myosin light chain isoform expression while reducing fetal troponin T phosphorylation, illustrating the impact of structural cues on proteoform-level maturation (Wilson et al., 2025).

Electrospun fibers incorporating conductive materials such as PEDOT:PSS, reduced rGO, gold nanorods, or MXene support electrical coupling and synchronous contraction of hiPSC-CMs, while simultaneously promoting sarcomere alignment and transcriptional maturation (Jang et al., 2025; Taylor et al., 2024; Lu et al., 2025). Combining conductive fibers with aligned topographies enhances both structural and electrophysiological maturation. In particular, conductive hydrogels embedding gold nanorods mimic native myocardial conductivity, which, when paired with co-culture systems or genetic interventions like CCND2 overexpression, further accelerates CM development (Jang et al., 2025).

Mechanical stimulation represents another complementary cue. Cyclic stretch applied to hiPSC-CMs improves sarcomere alignment, contractility, and calcium handling (Lu et al., 2025; Pohjolainen et al., 2022). Dynamic stiffening or strain-responsive substrates provide physiologically relevant mechanical environments that, when combined with aligned nanofibers, produce additive effects on both structural and functional maturation. More broadly, mechanical training platforms—whether static or cyclic—enhance ion channel expression, conduction velocity, and contractile rhythm stability, supporting the development of adult-like electrophysiological phenotypes.

*In vitro*, there is a marked mismatch between the stiffness of standard culture substrates and the ECM of physiological myocardium. Healthy human myocardium has a stiffness of 8–15 kPa, while diseased myocardium can reach 50–100 kPa depending on the pathology (Emig et al., 2021); while others claim that it can be up to 500 kPa (Lou et al., 2024). Standard tissue culture polystyrene (TCPS) plates have stiffness values in the 1–100 MPa range—several orders of magnitude higher than native myocardium (Hall et al., 2023; Emig et al., 2021). Excessive stiffness can have profound biological consequences. When the substrate modulus is far above physiological levels, CMs exhibit impaired contractility, disorganized sarcomeres, and reduced calcium transient amplitude. Stiff environments (>50 kPa) also activate CFs into myofibroblasts via mechanotransduction pathways such as integrin–FAK–RhoA/ROCK signaling, promoting fibrosis (Hall et al., 2023). Conversely, overly soft scaffolds may fail to provide adequate mechanical support for tissue organization and force transmission.

Physiologically tuned substrates can enhance CM maturation. HiPSC-CMs cultured on soft polydimethylsiloxane (PDMS) membranes with stiffness close to native myocardium matured faster, showing improved sarcomere organization and electrophysiological properties (Herron et al., 2016). Polyacrylamide hydrogels tuned to ∼10 kPa stiffness enhanced hPSC-CM contractile activity, calcium handling, and mitochondrial network organization (Ribeiro A. J. S. et al., 2015).

Scaffold stiffness can be tailored during fabrication. For example, atelocollagen scaffold baked at 150 °C for 150 min achieved ∼10 kPa stiffness, resembling healthy myocardium (Joanne et al., 2016). In PCL-based scaffolds, fiber alignment increased tensile modulus; however, hiPSC-CMs on aligned fibers exhibited better proliferation and more synchronized beating than on random fibers, likely due to anisotropic mechanical cues aligning cytoskeleton and gap junctions (Liu et al., 2023). Material blending can also modulate stiffness—incorporation of rGO or adjusting hydration state can soften stiff polymers (Tan et al., 2023). Silicone-PLGA patches with aligned fibers had localized stiffness of 13–20 kPa with bulk moduli of 350–750 kPa enhanced maturation and improved synchronization of calcium transients (Lou et al., 2024).

Surface functionalization of nanofibers with ECM proteins, plasma treatment, or peptide tethering further improves CM adhesion, sarcomere organization, and metabolic maturation. For example, engagement of collagen I via α2β1 integrins accelerates progenitor adhesion, proliferation, and differentiation, promoting adult marker expression and a metabolic shift toward fatty acid utilization (Barreto-Gamarra and Domenech, 2025). Co-culture with CFs supports ECM remodelling, cell alignment, and functional maturation; however, excessive fibroblast activity can compromise contractile performance, underscoring the importance of finely tuned microenvironments (Jang et al., 2025).

Genetic and pharmacological strategies can further complement scaffold-based approaches. Overexpression of genes such as CCND2 or Cfp1, as well as treatment with compounds like 3i-1262, promote sarcomere development, transcriptional remodelling, and metabolic shifts in hiPSC-CMs (Jang et al., 2025; Li et al., 2024; Pohjolainen et al., 2024). At the same time, the intrinsic dematuration plasticity of hiPSC-CMs highlights the need for stable, well-controlled culture conditions to maintain adult-like phenotypes (Meng et al., 2025).

Overall, these findings indicate that integrating topographical, electrical, mechanical, and biochemical cues within scaffold-based designs provides the most effective route to advanced hiPSC-CM maturation. Multi-modal strategies that combine aligned, conductive, and biochemically functionalized nanofibers with co-culture, genetic modulation, and metabolic optimization represent the current state-of-the-art in engineered cardiac tissue development (Lu et al., 2025; Taylor et al., 2024; Zhang et al., 2025).

### 4.3 Advanced coculture systems

Co-culture systems are gaining interest as they capture the cellular heterogeneity of the myocardium and provide stimuli for CMs that are absent in monocultures. By recreating interactions between CMs and non-myocyte cells, these models can promote structural, electrophysiological, and metabolic features that more closely resemble the adult heart.

**FIGURE 3:**
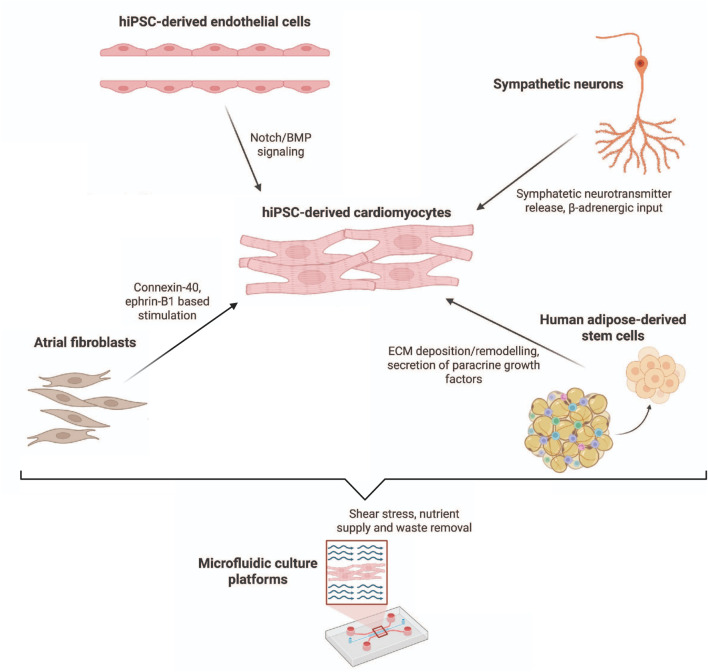
Co-culture approaches to support hiPSC-CM maturation. Non-myocyte partners—including endothelial cells, fibroblasts, adipose-derived stromal cells, sympathetic neurons, and integrated microfluidic systems—provide complementary biochemical and biomechanical cues that collectively drive structural, electrophysiological, and metabolic maturation of CMs.

Endothelial cells represent a key component of this strategy, as they engage in reciprocal communication with CMs. When co-cultured with hiPSC-CMs, they acquire cardiac-specific features while promoting sarcomere organization and contractility, partly through Notch- and BMP-mediated signaling (Helle et al., 2021). Embedding such co-cultures within 3D biomaterials, further enhances the expression of maturation markers and contractile gene networks, underscoring the benefit of combining cellular and structural complexity (Figure 3) (Gisone et al., 2025).

Fibroblasts also provide strong regulatory cues. In particular, atrial fibroblasts promote structural and electrophysiological maturation of hiPSC-derived atrial CMs via connexin-40 and ephrin-B1 signaling. Patterned co-culture systems integrating fibroblasts and CMs yield aligned tissue architecture with improved stability, enabling sophisticated applications such as atrial fibrillation modelling (Brown et al., 2024). Josvai et al. has also demonstrated that fibroblast-conditioned medium can reproduce many of the mechanical improvements seen in direct co-culture, although cell–cell contact remained essential for modulating rhythm and anisotropy (Josvai et al., 2025).

Stromal cells add another layer of support: human adipose-derived stem cells (hADSCs) improve ECM deposition and paracrine signaling, generating tissue sheets that not only display enhanced maturation *in vitro* but also improve engraftment *in vivo* (Zhang J. et al., 2024). In parallel, microfluidic organ-on-a-chip approaches have begun to integrate multiple supporting cell types. Liu et al. reported the development of a tri-culture system combining CMs, fibroblasts, and endothelial cells under perfusion, which enhanced ventricular marker expression, contractility, and adrenergic responsiveness compared to simpler formats (Liu et al., 2024). Finally, neuronal inputs have also been introduced, with sympathetic neurons shown to boost β-adrenergic signaling, calcium handling, and contractility, though without resolving metabolic immaturity (Mohammadi et al., 2025).

While these findings demonstrate the value of advanced co-culture strategies, important questions remain. The optimal cellular composition and ratios are still undefined, and different supporting cell types appear to promote distinct aspects of maturation. Reproducibility across laboratories, standardization of protocols, and integration with scalable platforms for pharmacological testing are further challenges. Future directions may involve combining multiple supporting populations in 3D engineered systems and systematically mapping which interactions are most critical for specific maturation endpoints.

## 5 Personalized medicine approaches

Patient-specific hiPSC-CMs offer a unique platform for precision cardiology by capturing individual genetic backgrounds and enabling mechanistic studies of CVD, including inherited arrhythmias and chemotherapy-induced cardiotoxicity (Burridge et al., 2016; Sala et al., 2022). By enabling such personalized assessments, hiPSC-CMs enhance the translational relevance of preclinical testing and support early-phase clinical decision-making. This capability underpins the concept of Clinical Trials in a Dish (CTiD), as discussed in recent perspectives, in which patient-derived cells are used to predict drug responses and guide pharmacogenomics-informed therapeutic decisions, reducing reliance on population averages (Fermini et al., 2018; Wu et al., 2024). The predictive power of these platforms is further strengthened by combining hiPSC-CM assays with high-throughput screening and machine learning, which enables prediction of proarrhythmic risk and drug-induced QT prolongation, tailored to individual genetic backgrounds (Serrano et al., 2023; Yoshinaga et al., 2019; Lee et al., 2024).

To refine these predictions, multi-omics, single-cell, and spatial transcriptomic analyses can be integrated with functional phenotyping. This combination allows detection of subtle patient-specific differences and supports scaffold-based high-throughput testing of individualized drug responses. In particular, biomimetic scaffolds enhance hiPSC-CM maturation, improving sarcomere alignment, contraction synchrony, and Ca^2+^ handling, which increases the physiological fidelity of patient-specific readouts compared with conventional 2D culture. When combined with HoC systems, these scaffolds reproduce key mechanical and electrical cues of the native myocardium, further bridging the gap between *in vitro* models and *in vivo* cardiac physiology (Wu et al., 2025; Kieda et al., 2024).

Finally, reliable patient-specific predictions depend on assay standardization. The Comprehensive *in vitro* Proarrhythmia Assay (CiPA) initiative has exemplified this principle by establishing standardized, multiparametric readouts–combining ion-channel data, *in silico* modelling, and hiPSC-CM assays–to improve the reproducibility and translational value of cardiotoxicity testing (Fermini et al., 2016; Konala et al., 2025). Reliable and standardized hiPSC-CM assays are crucial to enable the safe and effective application of these platforms in personalized clinical decision making.

In summary, the integration of patient-derived hiPSC-CMs with scaffold-based maturation strategies, multi-omics analyses, and standardized functional readouts create a comprehensive platform for precision cardiology. These systems enable mechanistic insight, predictive drug-response modelling, and individualized cardiotoxicity assessment, providing a robust foundation for both preclinical research and personalized clinical care.

## 6 Advanced platforms and integration

Recent innovations in the design and application of scaffolds have significantly enhanced their potential for cardiac tissue engineering, particularly through the integration of biomimetic systems that more closely replicate the complex structure and function of the native myocardium. Among the most important developments are capillary network mimicry, biomimetic hydrogels and perfusable culture systems, optical mapping, each contributing to improved CM viability, maturation, and function.

### 6.1 Hybrid scaffold systems

#### 6.1.1 Nanofiber-hydrogel composites

Hydrogels that mimic the mechanical and biochemical properties of native cardiac ECM are increasingly combined with electrospun nanofibers to form hybrid scaffolds. This strategy integrates the structural anisotropy and strength of nanofibers with the cell-friendly, hydrated 3D microenvironment of hydrogels, thereby supporting cell adhesion, proliferation, and electromechanical coupling. GelMA-based hydrogels, especially conductive composites such as gold-nanorod–GelMA or CNT–GelMA, promoted robust and synchronous beating of hiPSC-CMs, with enhanced cell–cell coupling and maturation. These effects are attributed to the formation of conductive pathways within the hydrogel that facilitate action potential propagation and calcium signaling (Zhang B. et al., 2024). Printable alginate/gelatin hydrogels filled with varying concentrations of carbon nanofibers yielded constructs with a Young’s modulus of 534.75 ± 2.7 kPa and a conductivity of 4.1 × 10^−4^ ± 2 × 10^−5^ S/cm. Although stiffer than native myocardium, the composite remained biocompatible with NIH/3T3 fibroblasts, highlighting its potential as a mechanically stable, electroconductive scaffold for cardiac tissue engineering (Serafin et al., 2021).

#### 6.1.2 Multi-layered architectures

A multilayered hybrid scaffold was developed by integrating a PCL nanofiber scaffold with fibrin and alginate hydrogels. The fibrin layer promoted angiogenesis, while co-culture of hADSCs and C2C12 myoblasts supported tissue regeneration. The resulting construct had a Young’s modulus of 33 ± 8.3 kPa—close to physiological myocardium—and maintained the survival and proliferation of hADSCs, HUVECs, and C2C12 cells. These properties suggest strong potential for supporting human CMs in EHT models (Kim D. et al., 2025).

#### 6.1.3 Perfusable systems with microchannel integration

While adequate pore size and porosity are crucial for nutrient diffusion and initial cell infiltration, pores alone cannot guarantee sufficient perfusion, especially in thicker cardiac constructs. Passive diffusion is limited to ∼100–200 μm, beyond which CMs become hypoxic and undergo cell death. This diffusion barrier represents one of the major limitations of engineered cardiac tissues. To address this, researchers have developed nanofiber-based scaffolds incorporating microchannels that mimic native capillary networks and can be perfused with culture media. These biomimetic channels not only enhance nutrient and waste exchange but also provide templates for endothelial cell lining, facilitating the formation of vascularized, functional cardiac tissues (Nwokoye and Abilez, 2024).

Fibrin-based tubular channels lined with endothelial cells supported oxygen perfusion and improved CM survival, leading to increased cell density and cross-sectional area (Vollert et al., 2013). Xiao et al. developed a strain-templated *cardiac biowire* system, in which CMs compacted around suspended tubing (50 µm ID) formed perfusable microchannels within engineered myocardium. This setup allowed perfusion with labeled particles, mimicking capillary transport, and enhanced nutrient delivery deep within the construct (Xiao et al., 2014). Microfabricated scaffolds patterned with endothelial cells have demonstrated the ability to form perfusable vascular networks *in vitro*, as shown by human embryonic stem cell (hESC)-derived ECs seeded in microchannel-patterned constructs (Redd et al., 2019). More recently, Paradiso et al. used 3D bioprinting combined with microfluidics-assisted coaxial wet-spinning to generate biomimetic cell-laden hydrogel microfibers. After 3 weeks, these constructs developed oriented, capillary-like networks within a fibrin-based core, demonstrating the feasibility of creating aligned microvasculature in engineered cardiac tissues (Paradiso et al., 2024).

Together, these examples highlight how hybrid nanofiber–hydrogel scaffolds can combine anisotropic structure, biochemical signaling, and tunable mechanics to more closely mimic native myocardium. By integrating conductive elements and angiogenic layers, such scaffolds show promise for drug testing platforms.

### 6.2 Sensing and monitoring platforms

To move beyond simple biocompatibility, EHT models must integrate sensing technologies for real-time response monitoring and allow for inducible disease conditions, making them powerful tools for drug testing and mechanistic studies. Recent advances have combined nanofiber scaffolds with microelectronic systems and lab-on-chip platforms to achieve these goals (Criscione et al., 2023).

#### 6.2.1 Microelectrode integration

A flexible PDMS beam-based microelectrode array (BMEA) integrated with a PCL nanofiber scaffold was developed for drug cardiotoxicity screening. The PCL nanofiber layer provided structural anisotropy and biocompatibility for hiPSC-derived CMs, supporting their attachment and maturation. The embedded microelectrode array enabled non-invasive, real-time electrophysiological monitoring, successfully detecting responses to varying concentrations of isoproterenol (ISO) (β-adrenergic agonist, increasing beat rate) and verapamil (calcium channel blocker, reducing contractility). This platform illustrates how nanofiber scaffolds can be functionally integrated with biosensors to evaluate pharmacological effects under physiologically relevant conditions (Zhang et al., 2025).

#### 6.2.2 Disease modeling platforms

A lab-on-a-chip platform combining PU nanofiber mats with controlled microenvironmental conditions was developed to simulate hypoxia. The nanofibers provided a biomimetic ECM substrate for human CMs and H2C9 cells, while integrated microfluidics enabled precise control of oxygen availability. Under hypoxia, cells exhibited hallmark disease phenotypes, including reduced ATP levels and downregulation of cardiac genes such as MAP4K, TNNT2, SERCA, and SCN5A, consistent with pathological remodeling seen in arrhythmia and heart failure models. Such systems highlight the potential of nanofiber–microfluidic hybrids for creating inducible disease states that are otherwise difficult to achieve in static culture (Kołodziejek et al., 2024).

Combining nanofiber scaffolds with sensing and microfluidic platforms paves the way for next-generation cardiac models that not only support maturation and functionality of hiPSC-CMs, but also provide real-time, multiparametric readouts, such as electrical activity, contractile force, calcium dynamics; high-throughput drug screening capability in standardized, miniaturized platforms and customizable disease modeling, including hypoxia, fibrosis, or arrhythmia induction (Liu B. et al., 2025). Ultimately, these hybrid models bridge the gap between simplified *in vitro* systems and *in vivo* physiology, offering versatile tools for predictive toxicology, drug discovery, and mechanistic studies of cardiac disease.

### 6.3 Rational design framework: reconciling engineering trade-offs

The evidence clearly indicates that no single parameter alone is sufficient for achieving complete CM maturation and functional cardiac tissue. Instead, successful tissue engineering requires sophisticated integration of physical, chemical, and biological cues (Table 5).

**TABLE 5:** Main parameters that should be integrated in EHT models. The table summarizes key structural, material, biological, electrical, and mechanical parameters relevant to EHT development, alongside their physiological relevance, impact on tissue maturation and function, and current technical approaches used to achieve these features.

Parameter	Physiological value	Impact	Technical capabilities	References
Fiber diameter	<500 nm	Important for cell adhesion and biocompatibility	Nanofibers (<500 nm)	Wittig and Szulcek (2021), Kai et al. (2011)
Material	Biocompatible, conductive, elastic	Important for cellular response and synchronized contractions	Hybrid materials, of scaffold (PCL, PLLA, PU, CS together with electroconductive fillers) in combination with hydrogels	Vogt et al. (2019), Ahamed et al. (2022), Parchehbaf-Kashani et al. (2020)
Fiber alignment	Aligned	Important for physical properties of scaffold, such as conductivity and stiffness, and critical for functional tissue organization, cell morphology, electrical coupling	Aligned fibers or micropatterned surfaces	Léger et al. (2024), Liu et al. (2023), Wilson et al. (2025)
Pore architecture	70% porosity, pore size 10–50 μm	Important for cell infiltration and nutrient transport and mechanical integrity of scaffold	Several techniques can increase porosity and vascularisation: electrospraying, structural tuning, thermally induced phase separation	Nwokoye and Abilez (2024), Flaig et al. (2024), Roshanbinfar et al. (2019), Fang et al. (2019), Mukasheva et al. (2024)
Coculture systems	CMs, mural cells (smooth muscle cells and pericytes), CFs, ECs and immune cells	Important for maturation through cell-cell interactions	Co-culture of CMs, CFs, ECs	Litviňuková et al. (2020), Jang et al. (2025), Brown et al. (2024), Josvai et al. (2025), Helle et al. (2021), Liu et al. (2024)
Biochemical factors	VEGF, EGF, FGF, IGF, PDGF, and ECM-derived peptides	Important for adhesion and maturation	ECM peptide incorporation, Growth factor immobilization in the scaffold or controlled release strategies	Brown A. L. et al. (2023), White and Chong (2020), Guex et al. (2014), Kang et al. (2022), Lakshmanan et al. (2016)
Electrical enhancement	Connexin-based gap junctions ensure conductivity of myocardium	Important for maturation and synchronization of contractions	Electroconductive fillers can improve electrical coupling (CNT, CQD, rGO, PPy, PANi)	Ahmadi et al. (2021), Mombini et al. (2019), Rastegar et al. (2021), Tan et al. (2023), Gu et al. (2018), Zarei et al. (2021), Ke et al. (2025)
Mechanical stimulation	10% contraction	Important for maturation	Elastic scaffold combined with mechanical stimulation	Kvam et al. (1997), Van Der Velden et al. (1998)

For instance, the multiscale 3D structure and anisotropic functional properties of cardiac tissue may require biofabrication strategies strategies, where few processes are combined to complement each other’s strengths and limitations [reviewed in Chansoria et al. (2021)]. The micropatterning techniques such as photolithography, microcontact printing, laser patterning may be employed to complement electrospinning for shaping and controlling cardiac cell alignment, anisotropy, sarcomere organization and vascularization.

The electrospun albumin nanofibrous membrane was laser-patterned to create aligned microgrooves for culturing neonatal CMs and obtained structure was combined with other differently patterned layers into thick 3D cardiac patches (Fleischer et al., 2017). The direct laser writing was used to create rectangular-shaped scaffolds for single-cell seeding to demonstrate the significant improvement of Ca^2+^ signaling properties in restructured iPSC-CMs (Silbernagel et al., 2020). The microcontact printing of patterned fibronectin provided a guidance cue for organization of seeded CMs and smooth muscle cells (Grosberg et al., 2012).

While the preceding sections have established that fiber diameter, alignment, conductivity, porosity, and stiffness each influence CM behavior, these parameters exist in a complex, interdependent design space where optimization of one property often compromises another. For instance, increasing fiber alignment enhances electrical anisotropy and sarcomere organization but reduces scaffold porosity and limits cell infiltration depth, while incorporating high concentrations of conductive fillers improves electrical coupling but may compromise mechanical integrity and biocompatibility. Rather than pursuing maximal values for individual parameters, effective scaffold design requires a systems-level approach that identifies which trade-offs are acceptable for a given application and implements architectures that mechanistically reconcile competing constraints. This framework provides a structured methodology for translating application-specific functional requirements—whether for high-throughput drug screening, disease modeling, or regenerative therapy—into rational scaffold design decisions that balance structural, electrical, mechanical, and biological objectives.

### 6.4 Computational modeling and AI-assisted scaffold design

Recent progress in computational modeling, artificial intelligence (AI), and *in silico* simulation is increasingly improving scaffold design and the development of cardiac drug-testing platforms. These approaches complement traditional experimental workflows by enabling rapid optimization of scaffold properties, more accurate prediction of scaffold–cell interactions, and improved reproducibility across batches and laboratories.

#### 6.4.1 AI-assisted and machine learning–driven scaffold optimization and mechanical/electrical property modeling

A growing number of evidence demonstrates that artificial intelligence (AI) and machine learning (ML) are becoming irreplaceable tools in scaffold engineering, including its mechanical and electrical properties. One of the earliest and most influential examples is the study by Bermejillo Barrera et al. (2021), in which 3D convolutional neural networks (3D-CNNs) were trained on virtual tomographic slices of CAD-generated scaffold geometries to predict key mechanical properties, including Young’s modulus, shear modulus, and porosity, prior to fabrication. This study demonstrated that scaffold architectures can be rapidly evaluated *in silico*, reducing the need for time-consuming experimental prototyping (Bermejillo Barrera et al., 2021). Another example is the explainable ML-based probabilistic framework, which uses reduced-order models to predict how scaffold geometry, material parameters, and fluid or mechanical loading collectively influence downstream cell differentiation outcomes (Drakoulas et al., 2024). These computational and ML-based strategies could be readily extended to electroconductive or anisotropic cardiac scaffolds, where integrated mechanical–electrical modeling would allow prediction of fiber-alignment effects, porosity-dependent conduction pathways, and deformation-induced changes in electrophysiology or contractile behavior before any physical construct is fabricated. A comprehensive review by Fu et al. (2025) highlights how ML algorithms are increasingly applied to predict and optimize a wide range of biomaterial and scaffold characteristics, from mechanical performance to degradation behavior, thereby accelerating development cycles and enabling more personalized or application-specific designs (Fu et al., 2025). In addition to that, the book chapter “Machine Learning Application in Tissue Engineering: Scaffold Design” by Shetty et al., underscores how predictive modeling can systematically link fabrication parameters, such as printing rate, layer thickness, or material formulation, to final scaffold outcomes, including porosity, compressive strength, and degradation kinetics. By establishing these quantitative relationships, ML-driven approaches support rational, data-guided scaffold design strategies that are far more efficient than traditional empirical or trial-and-error methods (Shetty et al., 2025). Together, these studies demonstrate how AI/ML methodologies are contributing to scaffold development for many tissues, including cardiac.

#### 6.4.2 Digital twins for scaffold–cell interaction prediction

The concept of a digital twin can be defined as a computational replica of a biological construct that updates dynamically as the physical system evolves. It is increasingly gaining attention in tissue engineering and regenerative medicine. In this context, a digital twin could integrate scaffold geometry, material degradation kinetics, and biological processes such as cell proliferation, ECM deposition, and electrophysiological maturation into a continuously evolving predictive model. While full digital-twin implementations for cardiac scaffold–cell systems are still in early development, advances in AI-assisted design, sensor-integrated culture platforms, and real-time imaging and functional readouts provide the necessary technological foundation. For example, the morphology-learning framework demonstrated simultaneous optimization of scaffold stiffness and predicted cell growth patterns, suggesting that similar models could eventually incorporate CM alignment, electrical conductivity, and contractile performance (Wang et al., 2024). Digital-twin strategies are already being deployed across tissue culture and musculoskeletal engineering, and even in cardiac electromechanical simulations, demonstrating their feasibility for predictive, personalized modelling of complex biological systems (Möller and Pörtner, 2021; Diniz et al., 2025). However, several major challenges in in silico twins application remain to realistically recapitulate cellular responses, and current developments are still struggling to address them. Firstly, evaluation of oxygen and nutrient transport and perfusion. Mathematical modeling must accurately describe oxygen and nutrient distribution within engineered cardiac tissue, particularly when using perfusion systems with parallel channel arrays and oxygen carriers to overcome diffusion limitations in thick constructs (Zingaro et al., 2023; Radisic et al., 2005; Rasouli et al., 2024). Secondly, mechanical stimulation integration: Cardiac tissue engineering requires proper incorporation of flow-induced dynamic effects and mechanical stimulation (compression, hydrodynamic pressure, fluid flow) into computational models, as these are critical modulators of cell physiology and tissue formation in cardiovascular applications (Jilberto et al., 2023; Thomas and Wu, 2025; Brown A. L. et al., 2023).

Finally, the overall multi-scale complexity has to be properly addressed to improve estimations of cellular responses *in silico*. The translation of biological knowledge on complex cardiac cell behavior, cell-cell and cell-ECM interactions, and tissue-specific microenvironmental gradients into predictive and robust engineering processes remains challenging, requiring integration of mechanistic understanding with computational tools that can capture both cellular and tissue-level phenomena (Thomas and Wu, 2025; Loerakker and Ristori, 2020).

Once adapted to scaffold-based cardiac constructs, digital twins would allow prediction of how material remodeling, mechanical loading, electrical stimulation or biochemical factors influence cell alignment, gap-junction maturation, and long-term tissue function.

## 7 Translational and regulatory perspectives in scaffold-based cardiac models

The translational deployment of scaffold-based cardiac tissues requires alignment with manufacturing and regulatory frameworks. Electrospinning for biomedical use must follow GMP-compatible principles, with controlled solvents, closed processing, and documentation of batch reproducibility, as highlighted in scale-up and technology reviews (Persano et al., 2013; Khorshidi et al., 2016; Mingjun et al., 2019; Dziemidowicz et al., 2021; Omer et al., 2021). Sterilization methods such as γ-irradiation, ethylene oxide, or aseptic processing must be selected based on their impact on polymer integrity and scaffold architecture (Holy et al., 2000; Dai et al., 2016; Horakova et al., 2020). Manufacturing standardization and batch-to-batch reproducibility are critical for regulatory acceptance. Variability arises from donor genetics, inconsistent differentiation protocols, scaffold fabrication tolerances, and fluctuating bioreactor conditions (National Academies of Sciences et al., 2019; Doulgkeroglou et al., 2020). GMP-compliant reagents (feeder-free, xeno-free media), automated liquid handling, and defined culture substrates reduce lot-to-lot variation and align with translational requirements (Humbert et al., 2025). Stirred suspension systems produce hiPSC-CMs with greater inter-batch consistency (∼94% purity, >90% viability post-cryopreservation) compared to adherent monolayer cultures (Prondzynski et al., 2024). Reproducible scaffold morphology remains essential (Pham et al., 2006; Lopez Marquez et al., 2022), in accordance with ISO 10993-1:2025, ASTM F2150-19, and ASTM F2900-11.

Because scaffold properties directly influence conduction, calcium handling, and contractile behaviour, scaffold-based cardiac constructs are increasingly relevant to regulatory cardiotoxicity assessment. The FDA’s CiPA (Comprehensive *in vitro* Proarrhythmia Assay) initiative defines electrophysiological endpoints for *in vitro* proarrhythmic risk using hiPSC-CMs with patch-clamp, optical mapping, and MEA platforms (Sager et al., 2014; Fermini et al., 2016; Blinova et al., 2018). Complementary EMA guidance, including the Reflection Paper on Stem Cell-Based Medicinal Products (2011), the ICH E14 QT/QTc guideline (2013), and the updated ICH E14/S7B Q&A (2022), emphasizes reproducible, mechanistic assays and standardized functional readouts. These requirements directly apply to scaffold-engineered tissues, whose architecture, stiffness, porosity, and surface chemistry strongly influence electrophysiological stability and thus QT/QTc-relevant endpoints. The FDA Modernization Act 2.0 permits the use of non-animal alternatives—including 3D cardiac constructs—in drug approval packages (Vicencio et al., 2024; Idais et al., 2025). Validation white papers co-authored by industry and academia outline standardized characterization principles, functional endpoints, and cross-platform benchmarking necessary for regulatory acceptance (Pang et al., 2019).

The application of scaffold-based cardiac models to drug screening requires systematic pharmacological validation beyond structural characterization. Functional assays must demonstrate reproducible, dose-dependent drug responses across multiple pharmacological classes. Engineered cardiac tissues cultured on 3D scaffolds show IC50 and EC50 values that align more closely with tissue-scale or clinical data compared to conventional 2D monolayers, reflecting enhanced maturity and physiological relevance (Marsano et al., 2016). However, the scaffold material itself can interfere with pharmacokinetic profiles through drug absorption, particularly affecting hydrophobic or hydrophilic compounds (Arai et al., 2020). This artifact must be minimized through careful material selection, preconditioning protocols, or scaffold-free alternatives when necessary. Benchmark comparisons against established systems are essential for validating novel platforms. Although adult human primary CMs offer high clinical predictivity, their limited scalability restricts broader adoption in early drug discovery (Pang et al., 2019). Microphysiological systems combining hiPSC-CMs, perfusion-based bioreactors, and multimodal readouts (contractility, electrophysiology, calcium transients) demonstrate potential for high-throughput cardiotoxicity screening with improved correlation to clinical outcomes (Marsano et al., 2016; Zhao et al., 2019).

Beyond single-organ approaches, translational relevance is further enhanced through multi-organ microphysiological systems capable of modelling systemic or metabolism-mediated cardiotoxicity, supported by multi-modal biosensing such as impedance, MEA, and optical mapping (Zhao et al., 2019; Ronaldson-Bouchard et al., 2022; Gu et al., 2024; Liu B. et al., 2025). Integrating multiple cardiac subtypes—such as atrial and ventricular CMs within a single construct—further enhances functional specificity and allows for chamber-specific drug responses (Zhao et al., 2019; Goldfracht et al., 2019). Importantly, scaffold-induced structural and functional maturation, reflected in improved sarcomere organization, conduction velocity, calcium cycling, and contractile force, enhances pharmacological predictivity. Aligned and electroconductive fibers guide CM orientation and synchronous excitation-contraction coupling (Kai et al., 2011; Bertuoli et al., 2019; Mancino et al., 2022). Biomimetic and piezoelectric substrates further promote electromechanical maturation (Hitscherich et al., 2016; Kitsara et al., 2022). Functional maturation correlates with improved detection of proarrhythmic liabilities, including QT prolongation, early afterdepolarizations, and conduction abnormalities, as demonstrated in engineered tissues and multicenter hiPSC-CM safety studies (Ribeiro M. C. et al., 2015; Blinova et al., 2018; Ronaldson-Bouchard et al., 2018; Goldfracht et al., 2019).

Standardized protocols for cell sourcing, scaffold characterization (fiber alignment, pore size, mechanical properties), and bioreactor operation are essential to ensure robust, reproducible preclinical drug screening platforms (Lind et al., 2017; Lopez Marquez et al., 2022). Cryopreservation of tissue-engineered products additionally enables high-throughput testing and large-batch quality assessments (Janssen et al., 2024; Laskary et al., 2025). As scaffold-based cardiac models mature, integrating quality-by-design principles, automated monitoring, and rigorous validation will be pivotal for their adoption in pharmaceutical pipelines and for reducing attrition rates caused by late-stage cardiotoxicity failures. Collectively, these advances position scaffold-based cardiac constructs as robust platforms for translational research and regulatory-aligned preclinical safety evaluation.

## 8 Technological barriers to standardization and reproducibility

Despite substantial progress in biomimetic nanofiber scaffolds for EHT models, manufacturing standardization and batch-to-batch reproducibility remain major barriers to their adoption in drug screening pipelines (Lamberger et al., 2025). Regulatory acceptance of *in vitro* cardiac platforms requires highly reproducible systems capable of delivering consistent structural and functional readouts across experiments, batches, and laboratories (EMA, 2025).

A key source of variability arises from the electrospinning process itself, where polymer properties, process parameters (electric field, polymer flow rate/feed rate, and printing speed) and environmental conditions (humidity, temperature, and atmospheric pressure) affect fiber diameter, alignment, porosity, and thickness thereby affecting cell attachment, alignment, electrophysiology, and calcium handling which are critical endpoints in drug testing assays (Shabani et al., 2025).

Material heterogeneity further complicates reproducibility. Natural and ECM-derived polymers (e.g., collagen, gelatin, fibrin) exhibit inherent lot-to-lot variability (Lin et al., 2025), while conductive composites may suffer from uneven dispersion, poor interface bonding or aggregation of fillers such as carbon nanotubes or graphene derivatives (Shar et al., 2023; Li et al., 2022). These inconsistencies can result in spatially heterogeneous mechanical and electrical properties, leading to variable cellular responses within and between scaffold batches.

Importantly, cell-related variability represents an additional and independent source of irreproducibility. Even when identical scaffolds are used, cell attachment efficiency and distribution can vary between experiments due to subtle differences in scaffold surface chemistry, protein adsorption leading to uneven specific cell adhesion sites, also handling protocols (Tran et al., 2010; Cámara-Torres et al., 2020). In 3D systems variations in initial cell attachment can propagate into differences in cell–cell coupling and functional maturation, ultimately influencing drug-response readouts such as beat rate, conduction velocity, and arrhythmic susceptibility. Furthermore, stem cell–derived CMs themselves exhibit batch-dependent heterogeneity in maturity, metabolic state, and electrophysiological properties, which can amplify scaffold-related variability (Jæger et al., 2025).

From a manufacturing perspective, scaling nanofiber-based EHTs to standardized, high-throughput formats remains challenging. Achieving uniform cell seeding across large or multiwell scaffolds, ensuring consistent tissue thickness, and integrating scaffolds with biosensors or microfluidic systems require precise and automated workflows. In addition, long-term culture stability, sterilization methods, and storage conditions can further influence both scaffold properties and cell behavior, yet are rarely standardized across studies.

Overall, while biomimetic nanofiber scaffolds offer significant promise for physiologically relevant cardiac drug testing, addressing both material- and cell-related sources of variability is essential to achieve the reproducibility and robustness required for regulatory acceptance.

## 9 Conclusion

**FIGURE 4:**
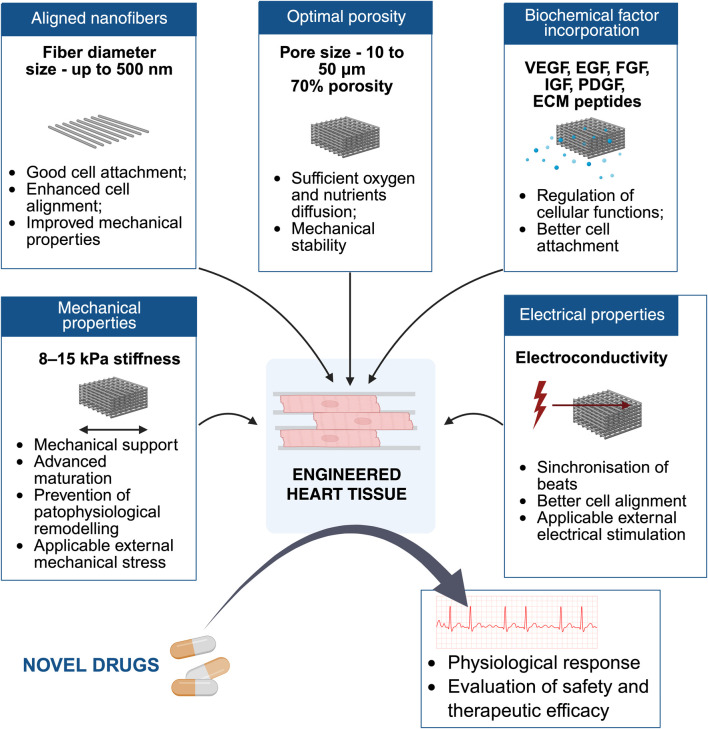
Key design parameters governing biomimetic electrospun scaffolds for engineered heart tissue (EHT) platforms. Aligned nanofibers, optimized porosity, tuned mechanical and electrical properties, and incorporation of biochemical factors collectively regulate CM organization, maturation, and electromechanical function. The resulting EHT enables physiologically relevant assessment of cardiac responses to novel drugs, including electrophysiological readouts for safety and efficacy testing.

Biomimetic scaffolds and electrospun nanofibers provide an excellent platform for CM culture and cardiac tissue engineering, offering unique capabilities for recapitulating native cardiac ECM architecture. However, successful practical translation requires careful optimization of fiber diameter (200–400 nm), material selection favouring conductive or bioactive polymers, appropriate fiber alignment, and balanced pore architecture for cell infiltration and nutrient transport (Figure 4).

Taken together, the field has made significant progress in understanding the individual contributions of various design parameters, but the integration of these factors remains challenging. Recent advances in conductive polymers and sophisticated coculture systems offer promising directions for addressing current limitations, particularly regarding CM maturation and electrical coupling.

This comprehensive review has synthesized recent developments in biomimetic scaffolds with a focus on their role as advanced platforms for drug testing and disease modeling. Future research should focus on developing more sophisticated hybrid systems that combine optimal physical properties with biological enhancement strategies. The ultimate goal remains the development of functional cardiac tissue that can effectively recapitulate native tissue responses in drug development platforms, thus accelerating the development of safer and more effective cardiac therapies or even integrate with native myocardium and restore cardiac function in patients with cardiovascular diseases.

While significant challenges remain, the continued advancement of biomimetic scaffold technologies and electrospun nanofiber systems, combined with improved understanding of CM biology, provides a strong foundation for future advanced applications in cardiac tissue engineering.

## Glossary

ADSC: Adipose-derived stem cell
CF: Cardiac fibroblast
CM: Cardiomyocyte
CNF: Carbon nanofiber
CNT: Carbon nanotube
CS: Chitosan
CVD: cardiovascular disease
Cx43: Connexin-43
D-PHI: Polar/hydrophobic/ionic polyurethane
EC: Endothelial cell
ECM: Extracellular matrix
EHT: Engineered heart tissue
EV: Extracellular vesicle
FBG–PLA: Fibrinogen–polylactic acid
FGF: Fibroblast growth factor
GelMA: Gelatin methacrylate
GO: Graphene oxide
HA: Hyaluronic acid
hiPSC-CM: Human induced pluripotent stem cell-derived cardiomyocyte
iPSC: Induced pluripotent stem cell
MHC: Myosin heavy chain
MLC: Myosin light chain
MMP: Matrix metalloproteinases
MSC: Mesenchymal stem cell
PANi: Polyaniline
PCL: Polycaprolactone
PDGF: Platelet-derived growth factor
PDMS: Polydimethylsiloxane
PEDOT: Poly(3,4-ethylenedioxythiophene)
PG: Poly(lactide-co-glycolide)
PLCL: Poly(lactide-co-ε-caprolactone)
PLGA: Poly(lactic-co-glycolic acid)
PPy: Polypyrrole
PSS: Polystyrene sulfonate
PU: Polyurethane
PVA: Polyvinyl Alcohol
rGO: Reduced graphene oxide
TGFβ: Transforming growth factor-β
TnT: Troponin T
VEGF: Vascular endothelial growth factor
